# Effect of Circadian Distribution of Energy and Macronutrients on Gestational Weight Gain in Chinese Pregnant Women

**DOI:** 10.3390/nu15092106

**Published:** 2023-04-27

**Authors:** Wenjuan Xiong, Shanshan Cui, Jia Dong, Yuanyuan Su, Yu Han, Zhiyi Qu, Shihao Jin, Zhi Li, Lei Gao, Tingkai Cui, Xin Zhang

**Affiliations:** 1 Department of Maternal, Child & Adolescence Health, School of Public Health, Tianjin Medical University, Tianjin 300070, China; 2 Beijing Key Laboratory of Environmental Toxicology, School of Public Health, Capital Medical University, Beijing 100069, China; 3 Department of Public Service, Tianjin Technician Institute of Mechanical & Electrical Technology, Tianjin 300350, China

**Keywords:** energy, macronutrients, circadian distribution, gestational weight gain

## Abstract

Gestational weight gain (GWG) may be affected by the timing of dietary intake. Previous studies have reported contradictory findings, possibly due to inconsistent characterizations of meal timing. We conducted a birth cohort study in Tianjin to determine the effect of daily energy and macronutrient distribution in mid and late pregnancy on GWG. Dietary intake information in the second and third trimesters used three 24-h dietary recalls, and meal timing was defined in relation to sleep/wake timing. The adequacy of GWG was assessed using recommendations from the Institute of Medicine guidelines. Pregnant women who had a relatively high average energy and macronutrient distribution in the late afternoon–early evening time window exhibited a greater GWG rate and a greater total GWG than that in morning time window during the third trimester (β = 0.707; β = 0.316). Carbohydrate intake in the morning of the second and third trimesters (β = 0.005; β = 0.008) was positively associated with GWG rates. Morning carbohydrate intake in the second trimester was also positively associated with total GWG (β = 0.004). Fat intake in the morning of the third trimester (β = 0.051; β = 0.020) was positively associated with the GWG rates and total GWG. Excessive GWG of Chinese pregnant women was related closely to eating behavior focused on the late afternoon–early evening and carbohydrate and fat intake in the morning during the second and third trimesters.

## 1. Introduction

Optimal Gestational weight gain (GWG) is essential to ensure the health of both the mother and the baby. However, GWG above or below the recommended guidelines of the Institute of Medicine is related to adverse perinatal outcomes, including gestational hypertension, gestational diabetes, cesarean delivery, premature birth, macrosomia, and infant mortality, as well as long-term negative outcomes in the offspring, including childhood obesity and adiposity [1,2]. Abnormal GWG is currently a serious obstetric issue. For example, in the United States, weight gained is higher than the Institute of Medicine-recommended range in 48% of women giving birth to full-term singleton infants, with 21% gaining insufficient weight [3]. In China, inadequate and excessive weight gain account for 27.2% and 36.6%, respectively [4]. On average, maternal weight increases as pregnancy progresses. The fastest weight gain occurs in the second trimester, and the weight gain rate in the third trimester slightly decreases [5]. In the second and third trimesters, weight gained includes maternal fat accumulation, extravascular fluid, placenta, uterus, and fetus growth [6]. Therefore, it is necessary to simultaneously pay attention to the GWG during the second and third trimesters and explore the associated factors. 

Emerging evidence suggests that the timing of food intake may affect weight gain. For instance, skipping breakfast, eating lunch late, and eating a large dinner have been associated with various indices of obesity [7]. Nevertheless, this subject has not been sufficiently studied in Chinese pregnant women, and one main methodological limitation is defining meal timing; conventional meal categories (i.e., breakfast, lunch, and dinner) and clock timing (external timing) to characterize meal timing may fail to accurately relate metabolic alterations in the context of the internal circadian rhythm [8]. Dim light melatonin onset is the recommended method for assessing the biological timing (internal circadian timing), which demands participants to stay in dim light conditions for a whole evening or more and undergo repeated blood or saliva collections to measure melatonin concentrations [9]. However, this method is unpractical for most epidemiological or clinical studies. A more practical approach to estimating the circadian time of food intake is to consider the timing of food intake relative to the sleep/wake cycle [10]. Moreover, changing an individual’s meal timing in a real-world setting may be difficult, but changing the daily distribution of energy or macronutrients may be achievable. Therefore, in the present study, we investigated dietary intake and sleep/wake timing in the second and third trimesters to define mealtimes relative to sleep/wake timing. We examined the associations between individual daily energy and macronutrient distribution, macronutrient intake in different time windows, and GWG. 

## 2. Materials and Methods

### 2.1. Study Design

Tianjin Maternal and Child Health Education and Service Cohort was a prospective cohort conducted at the Women and Children’s Medical Care Center in Hebei and Heping districts of Tianjin, China, beginning in January 2018. The inclusion criteria for the cohort were: (1) age ≥ 18 years; (2) singleton pregnancy; (3) in the first trimester (8–13 weeks) at enrollment; and (4) no plan to move from Tianjin during the subsequent 4 years. The participants in this study were a subsample of this ongoing cohort. The exclusion criteria were (1) having no Chinese speaking or reading abilities; (2) an individual history of diabetes, hypertension, liver failure, renal failure, congestive heart failure, abnormal thyroid function, psychosis, or cancer; and (3) a positive test result for women of COVID-19, syphilis, human immunodeficiency virus, rubella, toxoplasmosis, varicella, or cytomegalovirus [11]. Accordingly, the present analysis included 149 pregnant women who had complete data on at least two of the three visits for the study and had not been locked down during pregnancy. Six of these women were excluded because their daily average energy intake was <500 kcal/d or >3500 kcal/d (first-trimester visit and second-trimester visit: *n* = 5; first-trimester visit, second-trimester visit, and third-trimester visit: *n* = 1). Finally, 143 pregnant women were included (first-trimester visit and second-trimester visit: *n* = 34; first-trimester visit and third-trimester visit: *n* = 16; first-trimester visit, second-trimester visit, and third-trimester visit: *n* = 92) and 234 complete pieces of data for the participants were collected between 2018 January to 2021 December. 

### 2.2. Demographic Data and Covariates

In the first trimester, through face-to-face interviews, a self-administered questionnaire was used to collect the general demographic data of pregnant women, including age, educational level, employment status, and economic circumstances [12]. The height and weight within 1 month before pregnancy were self-reported. The pre-pregnancy body mass index (BMI) (weight (kg)/height(m)^2^) was calculated using pre-pregnancy weight and height [11]. Physical activity was measured once per trimester: first trimester, 8–14 gestational weeks; second trimester, 16–27 gestational weeks; and third trimester, 28–37 gestational weeks. Physical activity evaluation was conducted by asking the participants whether they had performed any physical exertion and the duration of daily physical activity (0 = “0 h/d;” 1 = “≤0.5 h/d;” 2 = “>0.5 h/d and ≤1.0 h/d;” 3 = “>1.0 h/d and ≤2.0 h/d;” and 4 = “>2.0 h/d”). Metabolic equivalents of the task were analyzed as reference thresholds of absolute intensities of the physical activities [13]. The pregnancy history, clinical history, gestational weight in each trimester, pre-delivery weight, and delivery condition were obtained from the women’s medical documentation in the Women and Children’s Medical Care Center. 

### 2.3. Estimation of GWG 

The evaluation indicators of GWG include total GWG across full pregnancy and the GWG rate in the second or third trimester.

First, the total GWG and the GWG rates were calculated as follows: 

The total GWG = pre-delivery weight (kg) − pre-pregnancy weight (kg);

$$The\;GWG\;rate=\frac{Weight\;at\;the\;last\;obstetrician\;visit(kg)-Weight\;at\;the\;first\;obstetrician\;visit(kg)}{Gestational\;age\;at\;the\;last\;obstetrician\;visit(w)-Gestational\;age\;at\;the\;first\;obstetrician\;visit(w)}$$

Second, to evaluate the adequacy of GWG according to the Institute of Medicine recommendation [14], the value of GWG in participants with different pre-pregnancy BMIs was reassigned by the recommended value. When GWG was within the range of recommended value (adequate): Values = 1; when GWG was below or above the recommended value:


$$\mathrm{Values}=(\frac{\mathrm{GWG}}{\mathrm{the}\;\mathrm{recommended}\;\mathrm{lower}\;\mathrm{limit}}+\frac{\;\mathrm{GWG}}{\mathrm{the}\;\mathrm{recommended}\;\mathrm{upper}\;\mathrm{limit}})/2$$

Values > 1 represent excessive, values < 1 represent insufficient [15]. 

### 2.4. Three 24-h Dietary Recalls

Through a five-stage multiple-pass interviewing technique, three 24-h dietary recalls were conducted by trained researchers to assess the dietary intake in the second and third trimesters [16]. To further reduce recall bias and improve accuracy, the trained researchers explained the recording requirements of dietary recalls to pregnant women a few days before the survey. They suggested taking notes or photos of the food they consume [17]. Three 24-h dietary recalls were performed over consecutive days, including one on the weekend. The evaluation of dietary intake composition did not consider nutrient supplementation. The number of eating episodes was ascertained by the number of caloric events ≥50 kcal, with time intervals between food consumption ≥15 min. Additionally, meal clock timing for each eating episode was recorded. The intake of energy and macronutrients was calculated using the average of the three 24-h dietary recalls by the software Yingyangjisuanqi v2.7.6.10, with the Chinese database as a reference. Energy intakes <500 kcal/d or >3500 kcal/d were excluded from the analysis. Daily energy and macronutrient (carbohydrate, protein, and fat consumption) distribution was calculated as a percentage of total energy and macronutrient intake and divided into four time windows, as mentioned previously.

### 2.5. Sleep/Wake Time and Daily Time Windows

At each visit, pregnant women were required to report their usual wake time, bedtime, and sleep onset latency on weekdays. Daily food intake for the participants did not occur during the habitual sleep period on weekdays; therefore, the analysis of time windows was concentrated on the waking period. We divided the waking period into four time windows based on the relationship between the internal circadian time and the sleep/wake cycle [8,18]. The “morning” time window was defined as within 2 h after getting up. The “late morning–early afternoon” time window was defined as from 2 h after getting up to the middle of the waking period. The “late afternoon–early evening” time window was defined as from the middle of the waking period until 2 h before bedtime, and the “night” time window was defined as within 2 h before bedtime.

## 3. Statistical Analysis

Pregnant women were classified into mutually exclusive dietary patterns by latent profile analysis. Latent profile analysis could identify unobserved heterogeneity in multiple continuous response variables. The Akaike information criterion (AIC), Bayesian information criterion (BIC), and sample-size-adjusted BIC (aBIC) were used to determine the best-fitting latent profile model. Additionally, the Vuong, Lo, Mendell, and Rubin likelihood ratio test was used to determine whether adding an additional profile contributed to a significantly better-fitting model [19].

For continuous variables, the Shapiro–Wilk test was used to assess the distribution of variables. Data with parametric distribution are described as mean and standard deviation and were compared using the one-way analysis of variance or *t*-test. Data with nonparametric distribution are described as median and interquartile range and were compared using the Kruskal–Wallis or Mann–Whitney U tests. For categorical variables, data are described as numbers (percentages) and were compared using the chi-squared test or Fisher’s exact test. Multiple comparisons were conducted using the Bonferroni post hoc test when necessary.

The Sample Wilcoxon Signed Rank Test was used to evaluate the adequacy of GWG in pregnant women with different dietary patterns. Spearman’s correlation was used to explore correlations among macronutrient intake in different time windows and the GWG. Multiple Linear Regression Models (method = backward) were used to determine the effects of dietary patterns (independent variables) and macronutrient intake in different time windows (independent variables) on the adequacy of weight gain (dependent variable). Models were adjusted for age, educational level, employment status, household income, pregestational BMI, parity, the condition of gestational diabetes mellitus, gender of offspring, physical activity, daily sleep duration, time node of the time window, number of eating episodes, total energy intake, and gestational week of delivery. *p* < 0.05 was considered statistically significant. Statistical analyses were performed using SPSS version 24 and Mplus version 6.0.

## 4. Results

### 4.1. Dietary Patterns Based on Energy and Macronutrient Distribution

The model fit information for latent profile analysis model estimation based on one to five latent profiles is shown in Table S1. The Vuong, Lo, Mendell, and Rubin likelihood ratio test did not indicate that the data in the four-class model fit were significantly better than that in the three-class model (*p* = 0.227). However, as the number of latent profiles was raised, the values of AIC, BIC, and aBIC were reduced, and the entropy remained above 0.80. Based on the results of model fit tests, our research objective, and the goal of simplicity, the four-class model was identified as the best description of latent dietary profiles.

**Figure 1:**
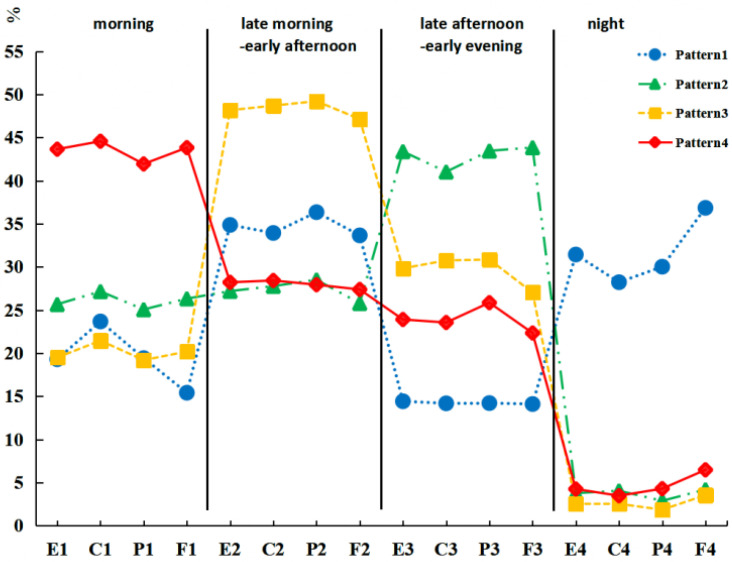
Average energy and macronutrient distribution of time windows in different dietary patterns. Latent profile analysis. E, energy; C, carbohydrate; P, protein; F, fat.

The four latent dietary profiles were characterized by average energy and macronutrient distribution in different time windows. Complete data of dietary recalls and sleep/wake time, 6.8% (*n* = 16, N = 234) were classified as having pattern 1, “high night distribution”. This group had a relatively high average energy and macronutrient distribution in the night time window. A total of 40.6% (*n* = 95, N = 234) were classified as having pattern 2, “high late afternoon–early evening distribution”. This group had relatively high average energy and macronutrient distribution in the late afternoon–early evening time window. Further, 31.2% (*n* = 73, N = 234) were classified as having pattern 3, “high late morning–early afternoon distribution,” who had relatively high average energy and macronutrient distribution in the late morning–early afternoon time window, and 21.4% (*n* = 50, N = 234) were classified as having pattern 4, “high morning distribution,” who had relatively high average energy and macronutrient distribution in the morning time window (shown in Figure 1).

### 4.2. Participant Characteristics

The sociodemographic and anthropometric characteristics and the dietary pattern composition did not appear to differ meaningfully across subsequent analyses (shown in Table 1).

Age, household income, eating episodes, and time node of the “early evening/night” time window of the pregnant women differed meaningfully by dietary profile in the second trimester. Age and time node of the “early evening/night” time window of the pregnant women also differed meaningfully by dietary profile in the third trimester (shown in Table 2).

Daily energy and macronutrient intake did not differ significantly by dietary profile in the second and third trimesters. The energy and macronutrient intake in different time windows differed significantly by dietary profile in the second and third trimesters (shown in Table 3 and Table 4).

In the second trimester, there were 14.3% (*n* =18) of pregnant women with insufficient GWG rates, 33.3% (*n* = 42) with adequate GWG rates, and 52.4% (*n* = 66) with excessive GWG rates. In the third trimester, there were 22.2% (*n* = 24) of pregnant women with insufficient GWG rates, 24.1% (*n* = 26) with adequate GWG rates, and 53.7% (*n* = 58) with excessive GWG rates. There were 23.9% (*n* = 22) of pregnant women with insufficient total GWG, 45.7% (*n* = 42) with adequate total GWG, and 30.4% (*n* = 28) with excessive total GWG.

### 4.3. The Adequacy of GWG in Pregnant Women with Different Dietary Patterns

**Figure 2:**
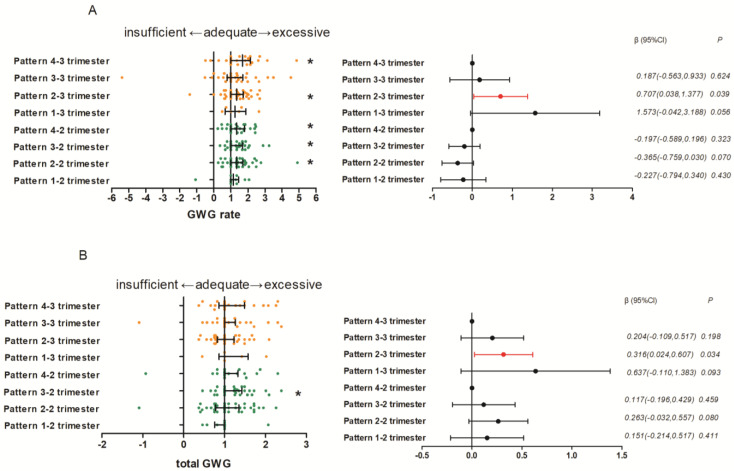
The effect of dietary patterns on GWG One Sample Wilcoxon Signed Rank Tests; Multiple Linear Regression Models (method = backward): (**A**) the effect of dietary patterns in the second and third trimesters on the adequacy of GWG rate; (**B**) the effect of dietary patterns in the second and third trimesters on the adequacy of total GWG * Values were significantly different from 1.

Pregnant women with a high late afternoon–early evening distribution in the second (Median (IQR) = 1.31 (0.70), Z = 3.391, *p =* 0.001) and third trimesters (Median (IQR) = 1.34 (0.72), Z = 3.065, *p* = 0.002), pregnant women with high late morning–early afternoon distribution (Median(IQR) = 1.00(0.68), Z = 3.296, *p =* 0.001) in the second trimester, and pregnant women with high morning distribution in the second (Median(IQR) = 1.35 (0.81), Z = 2.838, *p* = 0.005) and third trimesters (Median (IQR) = 1.68 (1.14), Z = 2.374, *p* = 0.018) appeared to have excessive GWG rates. Pregnant women with a high late morning–early afternoon distribution in the second trimester (Median (IQR) = 1.00 (0.31), Z = 2.374, *p* = 0.018) appeared to have excessive total GWG (shown in Figure 2).

### 4.4. Correlations between Macronutrient Intake in Different Time Windows and the GWG

**Figure 3:**
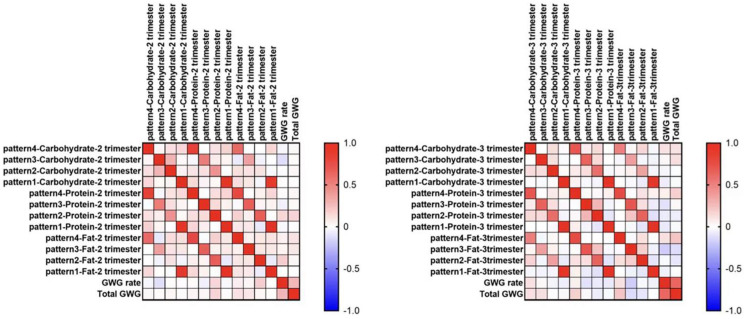
Correlations between macronutrient intake in different time windows and the GWG. Spearman’s correlation.

Fat consumption in the late afternoon–early evening of the second trimester was significantly positively correlated to the GWG rate of the second trimester (Spearman γ = 0.192, *p* = 0.031), fat consumption in the morning of the third trimester was significantly positively correlated to total GWG (Spearman γ = 0.220, *p* = 0.022) (shown in Figure 3).

### 4.5. Effect of Dietary Patterns and Macronutrient Intake in Different Time Windows on GWG

In the second trimester, carbohydrate (β (95% CI): 0.004 (0.000, 0.008); *p* = 0.043), fat (β (95% CI): 0.023 (0.010, 0.036); *p* = 0.001), and protein intake (β (95% CI): 0.015 (0.005, 0.026); *p* = 0.005) in the late afternoon–early evening time window, protein intake in the late morning–early afternoon time window (β (95% CI): 0.016 (0.006, 0.027); *p* = 0.003), and carbohydrate intake in the morning time window (β (95% CI): 0.005 (0.001, 0.010); *p* = 0.018) were positively associated with the GWG rates. Carbohydrate intake in the morning time window (β (95% CI): 0.004 (0.001, 0.007); *p* = 0.005) was positively associated with total GWG, and protein intake in the morning time window (β (95% CI): −0.014 (−0.026, −0.002); *p* = 0.022) were negatively associated with total GWG.

In the third trimester, fat intake in the late morning–early afternoon time window (β (95% CI): −0.023 (−0.044, −0.001); *p* = 0.041) and protein intake in the late afternoon–early evening (β (95% CI): −0.034 (−0.059, −0.010); *p* = 0.007) and morning time window (β (95% CI): −0.042(−0.073, −0.012); *p* = 0.007) were negatively associated with the GWG rates. Carbohydrate (β (95% CI): 0.008 (0.000, 0.016); *p* = 0.037) and fat (β (95% CI): 0.051 (0.017, 0.085); *p* = 0.004) intake in the morning time window was positively associated with the GWG rates. Pregnant women who had a relatively high average energy and macronutrient distribution in late afternoon–early evening time window exhibited a greater GWG rate than in morning time window (β (95% CI): 0.707(0.038, 1.377); *p* = 0.039) (shown in Figure 2). Protein intake in the late afternoon–early evening time window (β (95% CI): −0.013 (−0.024, −0.001); *p* = 0.028), carbohydrate intake in the late morning–early afternoon time window (β (95% CI): 0.004 (0.001, 0.007); *p* = 0.014), and fat intake in the morning time window (β (95% CI): 0.023 (0.011, 0.035); *p* < 0.001) were significantly associated with total GWG. Pregnant women who had a relatively high average energy and macronutrient distribution in late afternoon–early evening time window exhibited a greater total GWG than that in morning time window (β (95% CI): 0.316(0.024, 0.607); *p* = 0.034) (shown in Figure 2).

## 5. Discussion

To the best of our knowledge, the present study is the first to be conducted on Chinese pregnant women to investigate the effects of daily energy and macronutrient distribution on GWG during the second and third trimesters.

Our study findings showed that the proportion of pregnant women with an inadequate, adequate, or excessive trimester-specific mean rate of GWG (14.3%, 33.3%, 52.4% in the second trimester; 22.2%, 24.1%, 53.7% in the third trimester) and total GWG (23.9%, 45.7%, 30.4%) was approximately similar to some studies [4,20,21,22]. However, it differed from a large retrospective cohort study conducted with Chinese singleton pregnant women with gestational diabetes mellitus [23]. This large population-based study was conducted with Chinese singleton pregnant women who delivered between January 2011 and December 2017 in Beijing [24]. However, this could be due to the differences between populations, such as physical conditions and regional dietetic culture.

Though neither daily energy intake nor physical activity differed significantly across all dietary patterns in the present study, we found that pregnant women with the high late afternoon–early evening distribution in the second and third trimesters appeared to have excessive GWG rates; macronutrient (carbohydrate, fat, and protein) intake in the late afternoon–early evening time window of the second trimester was associated with greater GWG rates. Moreover, pregnant women who had a relatively high average energy and macronutrient distribution in late afternoon–early evening time window exhibited a greater GWG rate and a greater total GWG than in the morning time window during the third trimester. These findings are consistent with other studies conducted in pregnant women [15,25] and non-pregnant adults [8,26,27], which supported that higher intake in the evening was associated with a higher risk of weight gain. A potential mechanism may be associated with circadian changes in total energy expenditure, including resting metabolic rate and the thermic effect of food [28]. Randomized crossover trials reported that the endogenous circadian rhythm in the total energy expenditure of healthy adults peaked in the biological morning or early afternoon and was lower in the biological evening [29,30]. If the total energy expenditure was reduced, coupled with high energy-dense food intake, it might cause a positive energy balance in pregnant women as a contributing factor for excessive weight gained.

Additionally, we found that pregnant women with high late morning–early afternoon distribution in the second trimester with high morning distribution in the second and third trimesters experienced excessive GWG rates or excessive total GWG. Protein intake in the late morning–early afternoon time window of the second trimester, carbohydrate intake in the morning time window of the second trimester, carbohydrate intake in the late morning–early afternoon time window of the third trimester, and carbohydrate and fat intake in the morning time window of the third trimester was associated with greater GWG rates or greater total GWG. This finding is inconsistent with other studies conducted on pregnant women [15,25] and non-pregnant adults [8,26,27,31], which supported that higher morning or lunch intake was associated with a lower risk of weight gain. One possible reason is the difference between Chinese and Western food cultures. Taking breakfast as an example, nearly 90% of Chinese ingested cereals and tubers products (rich in carbohydrates), approximately 50% ingested vegetables, fruits, meat, fish, eggs, and milk, and only approximately 30% ingested beans and nuts [32]. In our study, participants with high morning distribution or high late morning–early afternoon distribution did not have a better diet quality for fruit components, milk, and nuts [33,34]. There is no significant difference in micronutrient intake across four dietary patterns (shown in Table S2). Much deep-fried food (rich in carbohydrates and fat) belongs to traditional Chinese breakfasts, such as dough sticks [35]. A previous study showed 4–8 weeks of overfeeding healthy adults with a high-fat breakfast resulted in 2–4 kg of weight gain [36], and a high-fat breakfast did not change satiety a few hours after breakfast [37]. Additionally, an increase of 1 g of carbohydrates was related to an increment of 17 g in weight during pregnancy. In comparison, 1 g of sugar was associated with an increase of 26 g of weight during pregnancy [38].

Interestingly, we found protein intake in the morning time window of the second trimester, protein intake in the late afternoon–early evening and morning time window of the third trimester, and fat intake in the late morning–early afternoon time window of the third trimester were negatively associated with the GWG rates or total GWG. This could be because foods high in protein are typically less energy dense. In healthy women, high-protein intake has a greater effect on satiety and appetite control and less subsequent food intake [39]. Though there is no significant difference in micronutrient intake across four dietary patterns, participants in our study were accustomed to eating food rich in monounsaturated and polyunsaturated fatty acids as snacks in the late morning–early afternoon time window. For instance, in nuts and yogurt, monounsaturated and polyunsaturated fatty acids have been found to contribute to weight loss and obesity prevention [40]. The total GWG was related more closely to eating behavior during the third trimester. This could be due to nonmonotonic fetal growth. However, the biparietal diameter and head circumference show an accelerated increase in the second trimester, while the abdominal circumference and estimated fetal weight velocity peak in the third trimester [41]. Another possible cause was the dietary counseling [42] after BMI monitoring in the second trimester; some pregnant women (65.2%, not shown in the result) in our study changed their dietary patterns after the second trimester.

This study has many strengths. First, we concurrently collected data about sleep timing and meal timing, which enabled us to establish an index indicating the circadian time of food intake. Second, we used a prospective design to assess multiple time points of sleep timing and dietary recalls, which reduced the potential effect of seasonal fluctuations in sleep timing and meal timing. Third, we considered the relationship between relative and absolute energy and macronutrient intake and GWG, since this is achievable in weight management during pregnancy. This study also has several limitations. The study was conducted on relatively healthy women in early pregnancy; therefore, the results of this study cannot be generalized to all pregnant women, especially those with high-risk pregnancies. A small sample size of pregnant women with higher night distribution hinders the observation of the association between the night distribution of energy and macronutrients and GWG. Moreover, a more detailed classification of nutrient consumption was not considered, such as saturated fatty acids, monounsaturated fatty acids, and polyunsaturated fatty acids. Future studies with more comprehensive investigations of sleep status and diet conditions in a larger population of pregnant women are needed.

## 6. Conclusions

Excessive GWG of Chinese pregnant women was related closely to eating behavior focused on the late afternoon–early evening time window and carbohydrate and fat intake in the morning during the second and third trimesters. Our findings emphasize that it is necessary to pay attention to Chinese pregnant women with high energy and macronutrient distribution in the late afternoon–early evening and adjust the macronutrient intake based on internal circadian timing for GWG management. Additionally, clinicians should provide more well-directed nutritional advice for pregnant women in different trimesters.

## Figures and Tables

**Table 1:** The characteristics of participants.

Variables	The Second Trimester (*n* = 126)	The Third Trimester (*n* = 108)	The Second and Third Trimester (*n* = 92)		F/χ^2^	*p*
	Mean ± SD/Median (IQR) or *n* (%)	Mean ± SD/Median (IQR) or *n* (%)	Mean ± SD/Median(IQR) Or *n* (%)			
Age (y)	30.85 ± 3.46	30.65 ± 3.63	30.88 ± 3.58		0.135	0.874
Level of education					0.328	0.988
High school or below	12 (9.52)	11 (10.19)	9 (9.78)			
College or university	101 (80.16)	88 (81.48)	75 (81.52)			
Master or higher	13 (10.32)	9 (8.33)	8 (8.70)			
Household income (thousand/y)					0.482	1.000
≤85	39 (30.95)	36 (33.33)	30 (32.61)			
≤180	60 (47.62)	48 (44.45)	43 (46.74)			
>180	27 (21.43)	24 (22.22)	19 (20.65)			
Unemployed					4.770	0.093
yes	15 (11.90)	14 (12.96)	4 (4.35)			
no	111 (88.10)	94 (87.04)	88 (95.65)			
Pregestational BMI (kg/m^2^)	21.50 (4.94)	21.50 (4.95)	21.45 (5.14)			0.979
Parity						
1	103 (81.75)	87 (80.56)	73 (79.35)		0.705	0.978
2	22 (17.46)	20 (18.52)	18 (19.56)			
3	1 (0.79)	1 (0.92)	1 (1.09)			
Gestational weeks	39.00 (2.00)	39.00 (2.00)	39.00 (2.00)			0.908
Gender of offspring					0.074	0.964
male	58 (46.03)	51 (47.22)	44 (47.83)			
female	68 (53.97)	57 (52.78)	48 (52.17)			
Gestational diabetes mellitus			The second trimester	The third trimester	0.459	0.928
Yes	21 (16.67)	18 (16.67)	15 (16.30)	18 (19.57)		
No	105 (83.33)	90 (83.33)	77 (83.70)	74 (80.43)		
Dietary Pattern			The second trimester	The third trimester	5.684	0.771
1	10 (7.94)	6 (5.56)	10 (10.87)	3 (3.26)		
2	51 (40.48)	44 (40.74)	35 (38.04)	40 (43.48)		
3	39 (30.95)	34 (31.48)	31 (33.70)	32 (34.78)		
4	26 (20.63)	24 (22.22)	16 (17.39)	17 (18.48)		

BMI, Body mass index; SD, Standard deviation.

**Table 2:** The sociodemographic, anthropometric, chronobiological, and personal characteristics of participants by dietary profile.

Characteristic	Dietary Pattern in the Second Trimester				*p*	Dietary Pattern in the Third Trimester				*p*
	1 (*n* = 10)	2 (*n* = 51)	3 (*n* = 39)	4 (*n* = 26)		1 (*n* = 6)	2 (*n* = 44)	3 (*n* = 34)	4 (*n* = 24)	
	Mean ± SD/Median (IQR) or *n* (%)	Mean ± SD/Median (IQR) or *n*(%)	Mean ± SD/Median (IQR) or *n*(%)	Mean ± SD/Median (IQR) or *n* (%)		Mean ± SD/Median (IQR) or *n* (%)	Mean ± SD/Median (IQR) or *n* (%)	Mean ± SD/Median (IQR) or *n* (%)	Mean ± SD/Median (IQR) or *n* (%)	
Age (y)	30.78 ± 2.83	31.76 ± 3.53 ^c^	29.62 ± 3.34 ^b^	30.91 ± 3.34	**0.035**	26.73 ± 3.43 ^bc^	30.88 ± 4.14 ^a^	31.23 ± 3.12 ^a^	30.37 ± 2.84	**0.039**
Level of education					0.841					0.515
High school or below	1 (10.00)	4 (7.84)	4 (10.26)	3 (11.54)		2 (33.33)	4 (9.09)	4 (11.76)	1 (4.17)	
College or university	9 (90.00)	43 (84.31)	30 (76.92)	19 (73.08)		4 (66.67)	37 (84.09)	27 (79.41)	20 (83.33)	
Master or higher	0 (0.00)	4 (7.84)	5 (12.82)	4 (15.38)		0 (0.00)	3 (6.82)	3 (8.82)	3 (12.50)	
Unemployed					0.447					0.074
yes	0 (0.00)	5 (9.80)	5 (12.82)	5 (19.23)		1 (16.67)	9 (20.45)	4 (11.76)	0 (0.00)	
no	10 (100.00)	46 (90.20)	34 (87.18)	21 (80.77)		5 (83.33)	35 (79.55)	30 (88.24)	24 (100.00)	
Household income(thousand/y)					**0.001**					0.215
≤85	3 (30.00)	14 (27.45)	10 (25.64)	12 (46.15)		4 (66.67)	15 (34.09)	12 (35.29)	5 (20.83)	
≤180	4 (40.00)	34 ^c,d^ (66.67)	15 ^b^ (38.46)	7 ^b^ (26.92)		1 (16.67)	23 (52.27)	13 (38.24)	11 (45.83)	
>180	3 (30.00)	3 ^c^ (5.88)	14 ^b^ (35.90)	7 (26.92)		1 (16.67)	6 (13.64)	9 (26.47)	8 (33.33)	
Pregestational BMI (kg/m^2^)	22.32 ± 1.20	23.01 ± 0.49	21.18 ± 0.48	22.74 ± 0.74	0.087	21.37 ± 1.18	21.99 ± 0.53	22.37 ± 0.58	23.38 ± 0.71	0.380
Parity					0.694					0.974
1	7 (70.00)	40 (78.43)	33 (84.62)	23 (88.46)		5 (83.33)	36 (81.82)	27 (79.41)	19 (79.17)	
2	3 (30.00)	10 (19.61)	6 (15.38)	3 (11.54)		1 (16.67)	7 (15.91)	7 (20.59)	5 (20.83)	
3	0 (0.00)	1 (1.96)	0 (0.00)	0 (0.00)		0 (0.00)	1 (2.27)	0 (0.00)	0 (0.00)	
Gestational weeks	40.00 (2.00)	39.00 (2.00)	39.00 (2.00)	39.00 (2.00)	0.293	39.50 (1.00)	39.00 (2.00)	39.00 (2.00)	39.00 (2.00)	0.855
Gender of offspring					0.175					0.258
male	7 (70.00)	20 (39.22)	16 (41.03)	15 (57.69)		2 (33.33)	17 (38.64)	17 (50.00)	15 (62.50)	
female	3 (30.00)	31 (60.78)	23 (58.97)	11 (42.31)		4 (66.67)	27 (61.36)	17 (50.00)	9 (37.50)	
Gestational diabetes mellitus					0.935					0.476
Yes	1 (10.00)	8 (15.69)	7 (17.95)	5 (19.23)		2 (33.33)	6 (13.64)	7 (20.59)	3 (12.50)	
No	9 (90.00)	43 (84.31)	32 (82.05)	21 (80.77)		4 (66.67)	38 (86.36)	27 (79.41)	21 (87.50)	
Physical activity (MET)	32.40 ± 1.48	28.73 ± 1.21	30.14 ± 1.14	28.85 ± 1.18	0.492	27.42 ± 2.84	32.06 ± 1.46	30.75 ± 1.45	31.35 ± 2.09	0.482
Daily sleep duration (h)	470.20 ± 18.20	508.45 ± 11.37	521.41 ± 10.33	525.73 ± 10.05	0.144	549.17 ± 25.18	530.75 ± 12.97	531.29 ± 13.01	524.67 ± 15.84	0.928
Eating episodes	4.70 ± 0.67 ^c,d^	4.10 ± 0.70	3.77 ± 0.77 ^a^	3.92 ± 0.80 ^a^	**0.004**	4.17 ± 1.33	4.25 ± 0.81	4.24 ± 0.70	4.00 ± 0.88	0.660
Time node of time window										
Morning/Late morning	09:05 ± 00:32	09:38 ± 01:00	09:49 ± 00:55	09:43 ± 00:58	0.621	10:12 ± 01:40	09:39 ± 00:49	09:41 ± 00:59	09:16 ± 00:51	0.178
Early afternoon/Late afternoon	14:25 ± 0:47 ^b^	15:01 ± 0:47 ^a^	15:33 ± 0:38	15:28 ± 0:53	**0.046**	14:57 ± 01:05	15:35 ± 00:38	15:33 ± 00:40	15:09 ± 00:52	0.362
Early evening/ Night	19:59 ± 01:24 ^b,c^	21:09 ± 00:50 ^a^	20:47 ± 00:42 ^a^	20:43 ± 01:01	**0.006**	19:22 ± 01:07 ^bcd^	20:56 ± 00:37 ^a^	20:58 ± 00:51 ^a^	20:29 ± 01:12 ^a^	**0.004**
Daily energy and macronutrients										
Energy (kcal)	1761.00 (1160.09)	1379.33 (451.00)	1477.00 (1052.80)	1721.50 (647.33)	0.318	1487.33 (1008.32)	1659.33 (684.08)	1586.92 (474.00)	1472.00 (498.33)	0.721
Carbohydrate (g)	226.60 (176.89)	185.80 (111.81)	234.63 (158.00)	218.72 (146.41)	0.538	178.00 (211.11)	238.18 (101.34)	237.38 (84.51)	222.05 (80.33)	0.810
Protein (g)	77.79 (50.07)	62.47 (30.27)	62.53 (41.77)	77.15 (33.28)	0.315	75.28 (35.64)	72.52 (24.98)	80.80 (39.08)	62.83 (31.58)	0.235
Fat (g)	59.07 (71.44)	44.77 (28.4)	40.03 (27.53)	46.44 (32.04)	0.335	55.68 (37.21)	45.90 (30.73)	42.96 (28.73)	37.52 (17.77)	0.372

^a^ Values were significantly different from pattern 1 (α level for statistical significance identified with Bonferroni correction). ^b^ Values were significantly different from pattern 2 (α level for statistical significance identified with Bonferroni correction). ^c^ Values were significantly different from pattern 3 (α level for statistical significance identified with Bonferroni correction). ^d^ Values were significantly different from pattern 4 (α level for statistical significance identified with Bonferroni correction). Significant tests shown in bold. BMI, Body mass index; MET, Metabolic equivalents of task; SD, Standard deviation.

**Table 3:** The differences in energy and macronutrient intake during the second trimester between each dietary pattern.

Nutrient	Dietary Pattern in the Second Trimester				*P* Value
	1 (*n* = 10)	2 (*n* = 51)	3 (*n* = 39)	4 (*n* = 26)	
	Median (IQR)	Median (IQR)	Median (IQR)	Median (IQR)	
**Energy and macronutrients intake in “morning” time window**					
Energy (kcal)	278.50 (509.74)	379.33 (221.33) ^d^	326.67 (218.67) ^d^	728.67 (369.17) ^b,c^	**<** **0.001**
Carbohydrate (g)	44.38 (80.55)	53.53 (37.24) ^d^	45.90 (38.25) ^d^	98.16 (77.74) ^b,c^	**<0.001**
Protein (g)	12.52 (22.40)	16.80 (10.64) ^d^	12.37 (8.09) ^d^	26.52 (13.88) ^b,c^	**<0.001**
Fat (g)	7.34 (15.64) ^d^	10.10 (7.04) ^d^	7.53 (9.26) ^d^	18.05 (11.28) ^a,b,c^	**<0.001**
**Energy and macronutrients intake in “late morning–early afternoon” time window**					
Energy (kcal)	505.00 (593.42)	407.00 (218.67) ^c^	674.00 (441.69) ^b,d^	456.00 (227.42) ^c^	**<** **0.001**
Carbohydrate (g)	61.93 (99.33)	51.90 (35.80) ^c^	96.93 (65.04) ^b,d^	56.94 (34.00) ^c^	**<0.001**
Protein (g)	30.50 (18.18)	17.60 (11.93) ^c^	28.27 (19.96) ^b,d^	19.47 (13.46) ^c^	**<0.001**
Fat (g)	15.85 (20.92)	10.57 (9.30) ^c^	19.53 (16.67) ^b,d^	12.72 (18.78) ^c^	**<0.001**
**Energy and macronutrients intake in “late afternoon–early evening” time window**					
Energy (kcal)	252.00 (395.38) ^b^	603.67 (290.89) ^a,c,d^	472.97 (385.00) ^b^	385.84 (175.19) ^b^	**<** **0.001**
Carbohydrate (g)	17.22 (58.32) ^b^	78.97 (74.77) ^a,c,d^	69.53 (45.17) ^b^	50.07 (32.06) ^b^	**<0.001**
Protein (g)	9.52 (16.88) ^b,c^	26.57 (14.40) ^a,d^	19.23 (16.30) ^a^	19.13 (9.07) ^b^	**<0.001**
Fat (g)	6.59(18.49) ^b^	16.17 (15.10) ^a,c,d^	9.90 (9.89) ^b^	8.96 (9.54) ^b^	**<0.001**
**Energy and macronutrients intake in “night” time window**					
Energy (kcal)	453.67 (535.25) ^b,c,d^	21.00 (106.67) ^a^	0.00 (36.00) ^a^	78.50 (167.75) ^a^	**<** **0.001**
Carbohydrate (g)	72.99 (87.08) ^b,c,d^	2.43 (15.87) ^a^	0.00 (4.90) ^a^	7.98 (15.32) ^a^	**<0.001**
Protein (g)	23.17 (30.44) ^b,c,d^	0.23 (2.77) ^a^	0.00 (0.93) ^a^	2.77 (6.72) ^a^	**<0.001**
Fat (g)	21.59 (25.21) ^b,c,d^	0.10 (2.57) ^a^	0.00 (0.77) ^a^	0.82 (6.4) ^a^	**<0.001**

^a^ Values were significantly different from pattern 1 (α level for statistical significance identified with Bonferroni correction). ^b^ Values were significantly different from pattern 2 (α level for statistical significance identified with Bonferroni correction). ^c^ Values were significantly different from pattern 3 (α level for statistical significance identified with Bonferroni correction). ^d^ Values were significantly different from pattern 4 (α level for statistical significance identified with Bonferroni correction). Significant tests shown in bold. IQR, interquartile range.

**Table 4:** The differences in energy and macronutrient intake during the third trimester between each dietary pattern.

Nutrient	Dietary Pattern in the Third Trimester				*p* Value
	1 (*n* = 6)	2 (*n* = 44)	3 (*n* = 34)	4 (*n* = 24)	
	Median (IQR)	Median (IQR)	Median (IQR)	Median (IQR)	
**Energy and macronutrients intake in “morning” time window**					
Energy (kcal)	440.67 (403.91)	389.84 (224.42) ^d^	333.50 (222.00) ^d^	633.67 (278.08) ^b,c^	**<** **0.001**
Carbohydrate (g)	61.77 (81.58)	56.92 (28.73) ^d^	48.18 (35.47) ^d^	86.48 (56.23) ^b,c^	**<0.001**
Protein (g)	21.85 (13.00)	17.65 (6.94) ^d^	15.28 (12.29) ^d^	25.40 (11.23) ^b,c^	**<0.001**
Fat (g)	8.72 (9.29) ^d^	10.70 (7.13) ^d^	9.15 (8.81) ^d^	19.12 (9.95) ^a,b,c^	**<0.001**
**Energy and macronutrients intake in“late morning–early afternoon” time window**					
Energy (kcal)	550.67 (460.50)	454.00 (229.25) ^c^	799.50 (282.25) ^b,d^	436.84 (266.17) ^c^	**<** **0.001**
Carbohydrate (g)	59.73 (92.64)	63.68 (35.83) ^c^	107.85 (43.50) ^b,d^	71.12 (50.54) ^c^	**<0.001**
Protein (g)	23.17 (7.71)	19.93 (12.59) ^c^	39.20 (22.83) ^b,d^	15.33 (15.85) ^c^	**<0.001**
Fat (g)	15.23 (19.43)	10.28 (14.11) ^c^	20.22 (13.62) ^b,d^	9.62 (10.77) ^c^	**<0.001**
**Energy and macronutrients intake in“late afternoon–early evening” time window**					
Energy (kcal)	156.83 (364.83) ^b^	714.00 (344.42) ^a,c,d^	464.50 (287.59) ^b^	373.67 (213.67) ^b^	**<** **0.001**
Carbohydrate (g)	17.40 (64.83) ^b^	91.91 (63.27) ^a,c,d^	56.59 (39.93) ^b^	56.02 (40.25) ^b^	**<0.001**
Protein (g)	7.27 (13.52) ^b,c^	29.29 (12.01) ^a,d^	23.28 (14.47) ^a^	20.02 (9.77) ^b^	**<0.001**
Fat (g)	3.05 (10.92) ^b^	16.95 (15.23) ^a,c,d^	10.58 (11.83) ^b^	8.55 (7.81) ^b^	**<0.001**
**Energy and macronutrients intake in “night” time window**					
Energy (kcal)	491.00 (294.33) ^b,c,d^	22.84 (127.33) ^a^	16.67 (104.75) ^a^	20.33 (97.83) ^a^	**<** **0.001**
Carbohydrate (g)	57.93 (40.38) ^b,c,d^	3.60 (17.28) ^a^	2.85 (10.84) ^a^	4.20 (10.26) ^a^	**<0.001**
Protein (g)	23.97 (17.53) ^b,c,d^	0.37 (3.55) ^a^	0.33 (3.92) ^a^	0.28 (3.88) ^a^	**<0.001**
Fat (g)	24.58 (11.18) ^b,c,d^	0.07 (3.36) ^a^	0.08 (2.88) ^a^	0.05 (4.20) ^a^	**<0.001**

^a^ Values were significantly different from pattern 1 (α level for statistical significance identified with Bonferroni correction). ^b^ Values were significantly different from pattern 2 (α level for statistical significance identified with Bonferroni correction). ^c^ Values were significantly different from pattern 3 (α level for statistical significance identified with Bonferroni correction). ^d^ Values were significantly different from pattern 4 (α level for statistical significance identified with Bonferroni correction). Significant tests shown in bold. IQR, interquartile range.

## Data Availability

The raw data analyzed in the present study will be available from the corresponding author on reasonable request.

## Supplementary Materials

The following supporting information can be downloaded at: https://www.mdpi.com/article/10.3390/nu15092106/s1, Table S1: Model fit information for latent profile analysis by number of estimated profiles; Table S2: The differences in micronutrient intake during the second and third trimester between each dietary pattern.
