# Supplementary material for: Effect of Circadian Distribution of Energy and Macronutrients on Gestational Weight Gain in Chinese Pregnant Women
Source: Nutrients. 2023 Apr 27;15(9):2106. doi: 10.3390/nu15092106 (PMC10181134; doi:10.3390/nu15092106)
Supplement: Supplementary file 1 [file nutrients-15-02106-s001.zip › Supplementary Table S1.pdf]

Table S1 Model fit information for latent profile analysis by number of estimated profiles

Latent profile	Model fit statistics					VLMR		
	**LL**	**AIC**	**BIC**	**a-BIC**	**Entropy**	**Mean**	**SD**	***P***
1	-14929.367	29922.734	30033.304	29931.879	—	—	—	—
2	-14453.518	29005.035	29174.346	29019.038	0.998	2.988	517.563	0.020
3	-14189.138	28510.276	28738.327	28529.137	0.908	6109.402	7408.418	0.920
4	-14004.790	28175.579	28462.371	28199.299	0.903	-5163.758	7377.273	0.227
5	-13849.154	27898.309	28243.841	27926.886	0.927	177.897	579.671	0.409

LL, log-likelihood; AIC, Akaike information criterion, BIC, Bayesian information criterion; a-BIC, sample-size-adjusted Bayesian information criterion; VLMR, Vuong -Lo-Mendell-Rubin likelihood ratio test.
