# Supplementary material for: Effect of Circadian Distribution of Energy and Macronutrients on Gestational Weight Gain in Chinese Pregnant Women
Source: Nutrients. 2023 Apr 27;15(9):2106. doi: 10.3390/nu15092106 (PMC10181134; doi:10.3390/nu15092106)
Supplement: Supplementary file 1 [file nutrients-15-02106-s001.zip › Supplementary Table S2.pdf]

Table S2 The differences in micronutrient intake during the second and third trimester between each dietary pattern

Nutrient	Dietary Pattern in the second trimester				*P* value	Dietary Pattern in the third trimester				*P* value
	1(n=10) Median (IQR)	2(n=51) Median (IQR)	3(n=39) Median (IQR)	4(n=26) Median (IQR)		1(n=6) Median (IQR)	2(n=44) Median (IQR)	3(n=34) Median (IQR)	4(n=24) Median (IQR)	
**Daily total nutrients/1000 kcal**										
Fiber(g)	12.45 (11.08)	8.90 (7.20)	8.80 (6.60)	12.70 (11.28)	0.203	10.55 (13.93)	11.55 (6.28)	10.00 (6.55)	8.75 (6.53)	0.756
Cholesterol (mg)	485.00 (215.50)	426.00 (252.00)	390.00 (235.00)	355.50 (311.50)	0.672	474.00 (484.00)	418.50 (247.75)	510.50 (342.75)	491.50 (291.50)	0.418
Calcium (mg)	446.50 (520.00)	450.00 (308.00)	464.00 (280.00)	528.50 (603.25)	0.754	550.50 (360.25)	482.00 (308.25)	502.50 (370.50)	451.00 (271.25)	0.927
Phosphorus (mg)	1009.55 (801.05)	873.00 (403.40)	960.50 (502.10)	1062.80 (430.75)	0.308	1030.15 (960.05)	973.85 (379.47)	1067.65 (478.4)	936.10 (304.88)	0.287
Potassium (mg)	2037.00 (1523.45)	1616.40 (820.30)	1692.80 (914.30)	1591.55 (1273.77)	0.583	1908.35 (1786.55)	1803.4 (639.65)	1931.10 (986.25)	1591.20 (973.05)	0.632
Sodium (mg)	936.55 (995.68)	804.60 (855.20)	746.60 (1196.30)	676.50 (909.61)	0.776	1070.60 (1696.88)	1017.4 (808.43)	1022.00 (915.97)	811.20 (407.67)	0.196
Magnesium (mg)	324.00 (230.75)	241.00 (109.00)	259.00 (166.00)	296.00 (202.75)	0.398	272.50 (337.25)	261.50 (134.00)	280.00 (142.75)	248.00 (156.75)	0.871
Iron (mg)	18.75 (21.85)	16.30 (8.80)	16.90 (7.90)	18.90 (18.38)	0.826	16.45 (15.83)	18.00 (7.88)	17.80 (10.05)	14.85 (8.43)	0.421
Iodine (μg)	39.60 (79.18)	26.90 (25.90)	27.10 (41.50)	33.70 (25.68)	0.604	34.45 (41.65)	29.00 (38.08)	32.50 (29.38)	35.30 (30.05)	0.969

Zinc (mg)	9.20 (5.86)	8.14 (4.06)	8.10 (4.02)	9.12 (5.11)	0.941	10.20 (7.35)	9.41 (2.93)	10.45 (4.25)	8.24 (3.37)	0.079
Selenium (μg)	44.41 (46.46)	46.94 (20.64)	44.05 (32.29)	49.86 (26.83)	0.667	55.01 (32.98)	53.06 (30.90)	60.35 (38.47)	48.11 (22.78)	0.251
Copper (mg)	1.60 (1.51)	1.33 (0.89)	1.36 (0.85)	1.53 (1.28)	0.493	2.03 (1.52)	1.42 (0.70)	1.66 (0.93)	1.27 (0.82)	0.172
Manganese (mg)	3.57 (2.67)	3.02 (1.86)	3.08 (1.57)	3.55 (3.81)	0.189	2.96 (4.01)	3.43 (1.89)	3.05 (1.94)	3.22 (1.66)	0.710
Vitamin A(μg RE)	417.50 (254.00)	312.00 (217.00)	286.00(248.00)	334.50 (315.75)	0.226	417.00 (259.50)	300.00 (175.75)	375.50 (297.50)	328.50 (190.50)	0.516
Vitamin D(μg)	1.40 (1.33)	1.40 (1.30)	1.60 (1.40)	1.75 (3.20)	0.932	1.35 (4.28)	1.30 (1.08)	1.45 (2.15)	1.80 (1.53)	0.357
Vitamin E(mg)	13.99 (22.02)	13.47 (9.77)	11.42 (9.78)	15.35 (13.02)	0.783	11.44 (12.47)	11.05 (11.71)	15.03 (8.59)	9.34 (11.78)	0.347
Thiamine (mg)	0.81 (0.83)	0.67[d] (0.37)	0.80 (0.56)	0.97[b] (0.54)	**0.020**	0.80 (0.64)	0.88 (0.41)	0.81 (0.36)	0.73 (0.33)	0.628
Riboflavin (mg)	1.13 (1.55)	0.87 (0.69)	0.86 (0.44)	0.84 (0.48)	0.411	1.14 (0.44)	0.86 (0.45)	0.98 (0.65)	0.94 (0.20)	0.708
Vitamin B6(mg)	0.24 (0.23)	0.15 (0.17)	0.16 (0.21)	0.19 (0.11)	0.646	0.22 (0.19)	0.18 (0.17)	0.17 (0.25)	0.17 (0.15)	0.782
Vitamin C(mg)	87.20 (107.60)	56.80 (44.10)	49.60 (49.20)	49.25 (48.43)	0.246	55.85 (123.52)	61.35 (57.45)	64.05 (54.95)	42.70 (66.43)	0.717
Folic acid(μg)	136.75 (153.60)	95.30 (79.00)	78.70 (107.60)	101.25 (87.48)	0.101	93.05 (141.05)	76.15 (80.43)	94.05 (133.35)	100.00 (103.00)	0.905
Nicotinic acid(mg)	16.09 (13.10)	13.35 (6.24)	12.91 (10.60)	12.66 (6.19)	0.759	16.41 (10.41)	13.63 (6.73)	15.57 (7.26)	11.62 (5.35)	0.085

[b] Values were significantly different from pattern 2 (α level for statistical significance identified with Bonferroni correction).
[d] Values were significantly different from pattern 4(α level for statistical significance identified with Bonferroni correction).
Signifificant tests shown in **bold**.
IQR, interquartile range
